# Loss-of-Function Variants in Cytoskeletal Genes Are Associated with Early-Onset Atrial Fibrillation

**DOI:** 10.3390/jcm9020372

**Published:** 2020-01-29

**Authors:** Oliver Bundgaard Vad, Christian Paludan-Müller, Gustav Ahlberg, Silje Madeleine Kalstø, Jonas Ghouse, Laura Andreasen, Stig Haunsø, Arnljot Tveit, Ahmad Sajadieh, Ingrid Elisabeth Christophersen, Jesper Hastrup Svendsen, Morten Salling Olesen

**Affiliations:** 1 Department of Biomedical Sciences, Faculty of Health and Medical Sciences, University of Copenhagen, 2200 Copenhagen N, Denmark; 2 Laboratory for Molecular Cardiology, Department of Cardiology, Centre for Cardiac, Vascular, Pulmonary and Infectious Diseases, Rigshospitalet, Copenhagen University Hospital, 2100 Copenhagen O, Denmark; 3 Department of Medical Research, Bærum Hospital, Vestre Viken Hospital Trust, 3004 Drammen, Norway; 4 Department of Clinical Medicine, Faculty of Health and Medical Sciences, University of Copenhagen, 2200 Copenhagen N, Denmark; 5 Department of Cardiology, Institute of Clinical Medicine, University of Oslo, 0450 Oslo, Norway; 6 Department of Cardiology, Copenhagen University Hospital, Bispebjerg, 2400 Copenhagen NV, Denmark; 7 Department of Medical Genetics, Oslo University Hospital, 0450 Oslo, Norway

**Keywords:** atrial fibrillation, genetics, arrhythmia, cardiology, next-generation sequencing, cardiomyopathy

## Abstract

Atrial fibrillation (AF) is the most common cardiac arrhythmia, and it is associated with an increased risk of heart failure, stroke, dementia, and death. Recently, titin-truncating variants (TTNtv), which are predominantly associated with dilated cardiomyopathy (DCM), were associated with early-onset AF. Furthermore, genome-wide association studies (GWAS) associated AF with other structural genes. In this study, we investigated whether early-onset AF was associated with loss-of-function variants in DCM-associated genes encoding cytoskeletal proteins. Using targeted sequencing, we examined a cohort of 527 Scandinavian individuals with early-onset AF and a control group of individuals free of AF (*n* = 383). The patients had onset of AF before 50 years of age, normal echocardiogram, and no other cardiovascular disease at onset of AF. We identified six individuals with rare loss-of-function variants in three different genes (dystrophin (*DMD*), actin-associated *LIM* protein (*PDLIM3*), and fukutin (*FKTN*)), of which two variants were novel. Loss-of-function variants in cytoskeletal genes were significantly associated with early-onset AF when patients were compared with controls (*p* = 0.044). Using publicly available GWAS data, we performed genetic correlation analyses between AF and 13 other traits, e.g., showing genetic correlation between AF and non-ischemic cardiomyopathy (*p* = 0.0003). Our data suggest that rare loss-of-function variants in cytoskeletal genes previously associated with DCM may have a role in early-onset AF, perhaps through the development of an atrial cardiomyopathy.

## 1. Introduction

Atrial fibrillation (AF) is the most common cardiac arrhythmia and affects more than 30 million individuals worldwide [1]. AF is associated with an increased risk of serious complications such as stroke, heart failure, dementia, and death [2]. At present, the disease represents a significant healthcare burden [3], and the prevalence is expected to increase more than two-fold in the coming decades [4]. The complex pathophysiological mechanisms behind AF initiation and maintenance are not yet completely understood. Current pharmacological treatments are limited in efficacy or associated with significant adverse effects [5]. AF also has a genetic component, which currently includes 166 common genetic variants [6] and rare variants in a variety of genes [7]. Whereas AF is traditionally regarded as an electrical disease, two recent studies identified loss-of-function (LOF) variants in the structural sarcomere gene titin (*TTN*) to be significantly enriched in early-onset AF patients [8,9]. In addition, several large genome-wide association studies (GWAS) associated structural genes with the disease [6,10], and an expert consensus document from several scientific societies suggested AF to overlap with an atrial cardiomyopathy [11].

There is substantial evidence that genes encoding cytoskeletal proteins are involved in skeletal muscle myopathies, many of which also have cardiac involvement [12]. Interestingly, the cytoskeletal gene *SGCG*, encoding the gamma-sarcoglycan protein, was recently associated with AF in a large GWAS [6]. *SGCG* was previously associated with skeletal muscle myopathies, and it is thought to be linked with dilated cardiomyopathy (DCM) [13]. The newly identified association between *SGCG* and AF suggests that cytoskeletal genes might play a role in AF, supporting the hypothesis of an atrial cardiomyopathy.

Here, we aim to assess the role of cytoskeletal genes in AF, by focusing on rare LOF variants in a highly selected cohort of AF patients with early onset of disease.

## 2. Materials and Methods

### 2.1. Study Populations

Our case population included 527 Danish and Norwegian patients with onset of AF at age < 50 and presenting with no other cardiovascular disease. The diagnosis of AF was defined by ICD-10 code I48. Patients with diabetes, hypertension, hyperthyroidism, congenital heart disease, valvular heart disease, or congestive heart failure were excluded. We also excluded patients with a left ventricular ejection fraction <55%, as determined by echocardiographic examination. Danish patients were identified through the National Danish Patient Registry, while Norwegian patients were identified and recruited through clinical practice and the Norwegian Atrial Fibrillation Registry. The study was approved by the Scientific Ethics Committee of Copenhagen and Frederiksberg (Protocol reference number: H-KF-01313322) and the Regional Ethics Committee (REK) in Norway (Protocol reference number: 2009/2224-5).

The control population consisted of 383 Danish individuals from the Copenhagen Holter Study. These individuals were monitored for arrhythmia with Holter monitors for 48 h and were known to be free of AF and cardiovascular disease. The cohort was previously described in detail elsewhere [14]. Characteristics of the study populations are summarized in Table 1. The control group was approved by the Scientific Ethics Committee of Copenhagen and Frederiksberg (Protocol reference number: KF 01 25304).

### 2.2. Genetic Sequencing

We extracted genomic DNA (gDNA) from leukocytes in peripheral blood samples from study participants in both case and control populations. The gDNA was fragmented with endonucleases and hybridized with gene-specific probes from the Illumina TruSight Cardio Sequencing Kit. The hybridized fragments were captured with magnetic beads, PCR-amplified, and sequenced using Illumina HiSeq 2500 and NextSeq technology. Reads were aligned to the Human Reference Genome (NCBI Genome Build 37) using the Burrow Wheelers Aligner algorithm [15], and post-processed in accordance with Genome Analysis Toolkit version 3.4 (GATK) guidelines [16].

### 2.3. Selection of Candidate Genes

We focused on genes encoding cytoskeletal proteins in cardiomyocytes, which were reported to be linked with DCM in previous literature [17]. Our candidate genes consisted of the following genes: *DMD* (dystrophin), *CRYAB* (crystallin alpha B), *DES* (desmin), *PDLIM3* (actin-associated *LIM* protein), *FKTN* (fukutin), *FKRP* (fukutin-related protein), *LAMA4* (laminin subunit alpha 4), and *SGCD* (delta-sarcoglycan).

### 2.4. Evaluation of Identified Variants

Annotation of variant coding DNA and consequence of variants were performed using the tool TransVar [18]. LOF variants were defined as variants leading to a premature stop codon in coding DNA, or variants affecting splice donor or splice acceptor sites. In genes producing multiple protein isoforms, we focused on isoforms expressed in atrial tissue. Isoform expression analysis was conducted using the online database The Genotype Tissue Expression Database version 8 (GTEx v8) [19].

### 2.5. Pathway Analysis

We performed a pathway analysis of protein-protein interactions, using tool STRING [20]. Pathway analysis was performed on the human protein products of all genes in which LOF variants were identified. The results were filtered, and interactions were assigned confidence scores. The methods for calculating these confidence scores were described in detail elsewhere [21]. For each gene, we focused on the ten protein-protein interactions with the highest confidence scores, and we excluded interactions with a confidence score <0.700.

### 2.6. Statistical Analyses

Due to the small sample size, we used Fisher’s exact test for comparisons of variants in patients versus controls. Using an LD regression score [19], we also examined the genetic correlation between AF and 13 other traits: alcohol dependence, angina, body mass index (BMI), coronary heart disease, depression, diabetes type 2, smoking, hand grip strength, heart failure, height, hypertension, non-ischemic cardiomyopathy, and overall health rating. These analyses were based on a similar analysis conducted in a previous study [22], modified to also include non-ischemic cardiomyopathy. The GWAS data were derived from publicly available summary statistics, summarized in Table S1 (Supplementary Materials). We applied Bonferroni correction to account for multiple testing, and *p* < 0.05/13 was considered significant. Statistical analyses were performed using the software R, version 3.6.0.

## 3. Results

### 3.1. Clinical Characteristics

We identified six patients with LOF variants in genes encoding cytoskeletal proteins. The clinical characteristics of the individuals with LOF variants are summarized in Table 2. Four Danish patients (I through IV) harbored LOF variants in *DMD*, whereas two Norwegian patients carried LOF variants in *FKTN* (patient V) and *PDLIM3* (patient VI), respectively.

All six patients had very early onset of disease; the maximum age at onset of disease was 40 years. All four patients with *DMD* variants had onset of AF before age 30 and were all diagnosed with persistent AF. Interestingly, five of the six patients were male, including all of the four patients with *DMD* variants, making them hemizygous for the variants. The individuals with LOF variants in *FKTN* and *PDLIM3* were both heterozygous for the variants.

Three of the six patients self-reported family history of AF (Table 2). These family members were unfortunately not available for genetic sequencing.

### 3.2. Genetic Variation

We identified four different LOF variants in three cytoskeletal genes. Two of the LOF variants were identified in *DMD*, one in *FKTN*, and one in *PDLIM3*.

Two Danish AF patients carried a variant in *DMD*, which affected the splice donor sites in multiple *DMD* isoforms expressed in cardiac tissue, notably isoform Dp427m (ENST00000357033) and isoform Dp71b (ENST00000378723). In Dp427m, it affected the donor splice site between exon 71 and exon 72, while affecting the donor splice site between exon 10 and 11 in Dp71b. Another variant in *DMD* was present in two other Danish patients. This variant resulted in a frameshift in several DMD isoforms, including the isoforms Dp71b (ENST00000378723) and Dp260 (ENST00000358062). We identified the variant *FKTN* p.Q54X that caused a stop codon in exon 3 in one Norwegian AF patient. Finally, one Norwegian individual harbored a frameshift variant in *PDLIM3* (p.C246*fs*1), resulting in a premature stop-codon in exon 6. The characteristics of all variants are summarized in Table 3. Variants affecting multiple protein isoforms are summarized in Table S2 (Supplementary Materials).

The association test showed a significantly larger proportion of LOF variants in cytoskeletal genes in early-onset AF patients compared with the controls free of AF (*p* = 0.044).

### 3.3. Genetic Correlation

**Figure 1:**
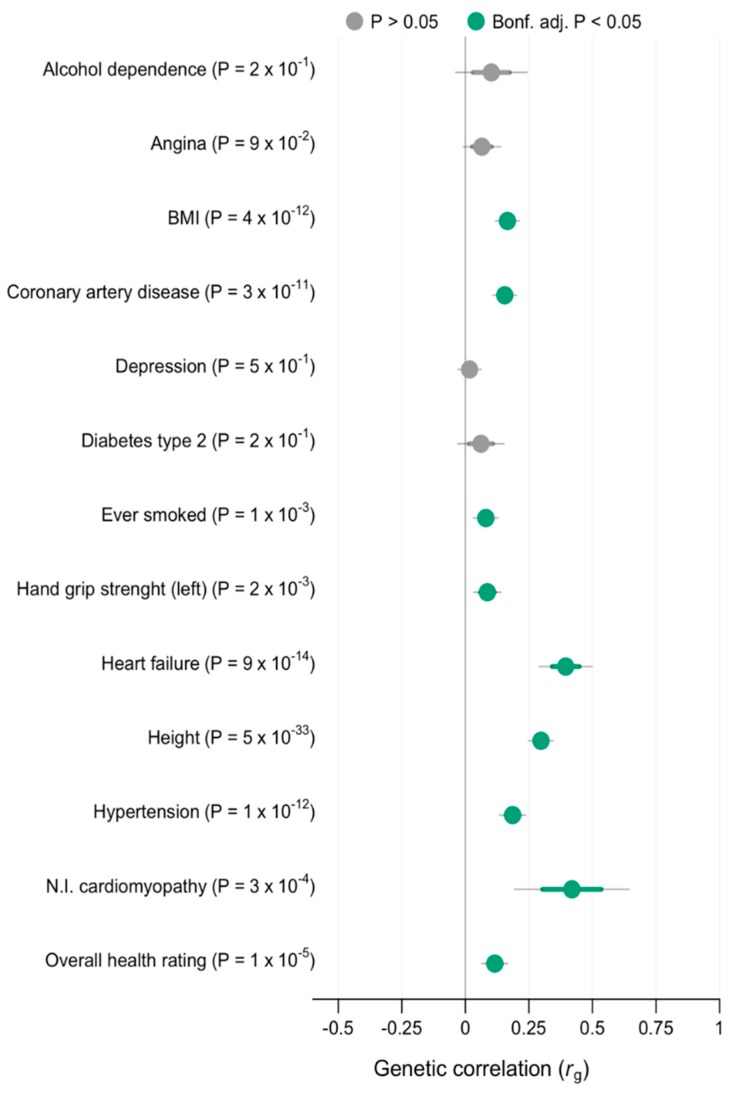
Genetic correlation between AF and 13 other traits with 95% and 99% confidence intervals. Correlations that were significant when accounting for multiple testing are marked in green. Bonf. adj., Bonferroni adjusted; BMI, body mass index; N.I. cardiomyopathy, non-ischemic cardiomyopathy.

We found a significant, moderate genetic correlation (r_g_ = 0.40–0.59) between AF and non-ischemic cardiomyopathy (r_g_ = 0.42 (SE = 0.12); *p* = 3 × 10^−4^). We also found a significant, small correlation between AF and heart failure (r_g_ = 0.39 (SE = 0.05); *p* = 9 × 10^−14^), height (r_g_ = 0.30 (SE = 0.02); *p* = 5 × 10^−33^), hypertension (r_g_ = 0.19 (SE = 0.02); *p* = 1 × 10^−12^) BMI (r_g_ = 0.17 (SE = 0.02); *p* = 4 × 10^−12^), coronary heart disease (r_g_ = 0.16 (SE = 0.02); *p* = 3 × 10^−11^), overall health rating (r_g_ = 0.12 (SE = 0.03); *p* = 1 × 10^−5^), smoking (r_g_ = 0.08 (SE = 0.03); *p* = 1 × 10^−3^), and hand grip strength (left hand) (r_g_ = 0.08 (SE = 0.03); *p* = 2 × 10^−3^). We found no significant genetic correlation between AF and alcohol dependence, angina, depression, and diabetes type 2. Genetic correlation results are illustrated in Figure 1.

### 3.4. Pathway Analysis

**Figure 2:**
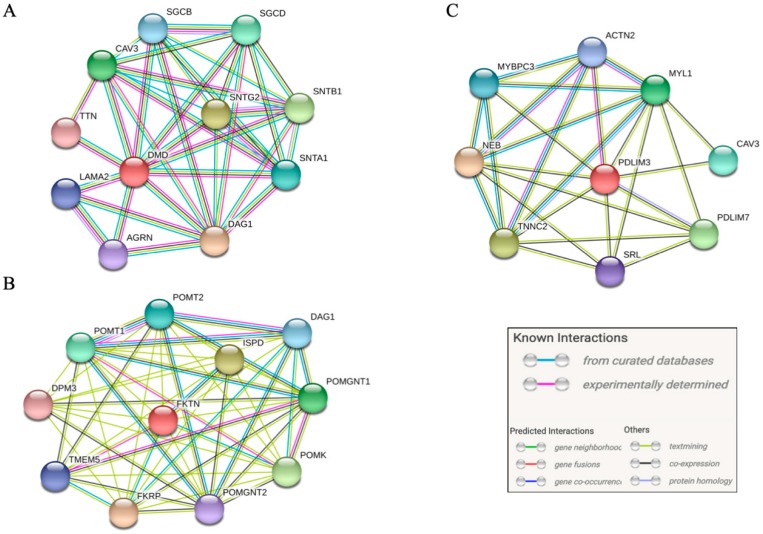
Protein-protein interactions with high confidence score (confidence score >0.7). (**A**) interactions of the product of *DMD*, (**B**) interactions of the product of *FKTN*, and (**C**) interactions of the product of *PDLIM3*.

We found interactions between the protein products of *DMD*, *FKTN*, and *PDLIM3* and numerous other genes. *DMD* had protein-protein interactions with more than 10 other genes, with the *TTN* gene among top ten interactions with highest confidence score. *FKTN* also interacted with more than 10 other genes, while *PDLIM3* had protein-protein interactions with eight other genes. The top ten protein-protein interactions with highest confidence scores for all three genes are summarized in Table S3 (Supplementary Materials). Interaction networks are illustrated in Figure 2.

## 4. Discussion

In this study, we identified four different LOF variants in three cytoskeletal genes (*DMD*, *FKTN*, and *PDLIM3*) carried by six individuals with early-onset AF. There was a significant enrichment of LOF variants in cytoskeletal genes in the case population compared to an arrhythmia-free control population, in which no individuals carried LOF variants.

### 4.1. Variants in DMD

Four Danish patients with early-onset AF carried two different LOF-variants in *DMD*. The variant-carriers had a considerable burden of disease; all four presented with very early onset of disease (age <30 years) and developed persistent AF (Table 3). Deleterious variants in *DMD* are associated with the serious muscular dystrophies Duchenne and Becker, in which skeletal muscle function is severely impaired along with serious cardiomyopathies [23]. However, none of the participants harboring variants in *DMD* were diagnosed with skeletal muscle dysfunction. *DMD* produces numerous isoforms [24], and predicting the effect of variants in the different isoforms on cardiac function is challenging. Several cases of *DMD*-associated, X-linked cardiomyopathy without a skeletal muscle phenotype were reported [25], which emphasizes the complex pathophysiology of *DMD* variants. Notably, all four individuals with *DMD* variants were male and, therefore, hemizygous for the variants. It is possible that these variants, while having arrhythmogenic effects in males, are less pathogenic in females who have a second copy of the *DMD* gene.

Of the two *DMD* LOF variants, the frameshift variant (p.D615Efs*6) did not affect the primary isoform expressed in skeletal and cardiac muscle, Dp427m. The variant did, however, affect several other isoforms expressed in cardiac tissue, including Dp71b (ENST00000378723), a product of alternative splicing of the ubiquitously expressed Dp71(24), and numerous other isoforms such as Dp260 (see Table S2, Supplementary Materials). Although the exact function of these isoforms in the heart is not yet known, the Dp71b isoform with the transcript ENST00000378723 is highly expressed in the atria (Figure S1, Supplementary Materials), and previous studies showed the Dp260 isoform to be expressed in cardiac tissue [26].

The *DMD* splice donor variant (c.10262+1G > A) affected numerous isoforms, including Dp427m, an isoform important for the function of skeletal and cardiac muscle function. Interestingly, this variant was previously reported in a patient who suffered from sudden cardiac death [27], which supports possible proarrhythmic effects of the variant. Studies of lethal cardiac diseases in recent years emphasizes that the pathogenicity of genetic variants in conserved genes may not be as malignant as previously thought [28,29]. It is acknowledged that rare variants have different disease-causing effects and that disease burden and severity are affected by a combination of rare and common variants, in addition to other non-genetic factors [30]. Therefore, the *DMD* splice donor variant may predispose to lethal arrhythmia in one individual while causing less lethal disease in another. The variant c.10262+1G > A had a minor allele frequency (MAF) of 0.027% in gnomAD, indicating that it is not a major contributor of highly malignant disease.

Additionally, pathway analysis (see Figure 2A) suggests protein-protein interactions between *DMD* and several other genes expressed in the heart. Notably, *DMD* interacts with *TTN,* a gene which was previously linked with AF [6,8].

### 4.2. Variants in FKTN and PDLIM3

We identified LOF variants in the cytoskeletal genes *FKTN* and *PDLIM3* in two Norwegian patients with early-onset AF. Both variants identified were novel and, to the best of our knowledge, not reported elsewhere.

*FKTN* is associated with the rare muscular dystrophy, Fukuyama-type muscular dystrophy [31]. However, *FKTN* variants were in some cases also linked with DCM with minimal skeletal muscle involvement [17,32], and our pathway analysis (Figure 2B) predicts protein-protein interactions between the product of *FKTN* and the product of *FKRP* (fukutin-related protein), among others. *FKRP* was previously associated with DCM [33].

Similarly, *PDLIM3* was also associated with both DCM [17,34] and muscular dystrophy, in the form of myotonic dystrophy [35]. The protein product of *PDLIM3* is predicted to interact with the protein products of several other genes that are linked with heart disease, including *MYBPC3*, *ACTN2*, and *CAV3* [13] (Figure 2C).

### 4.3. Atrial Cardiomyopathy

Our understanding of the pathophysiological disease mechanisms of disease-causing genetic variants changed in recent years. Evidence from large-scale GWAS and high-throughput sequencing projects [6,10] suggests that diseases, which previously were thought to have completely different underlying mechanisms, may be more closely related, and even part of a continuum. For instance, recent reports of an enrichment of titin-truncating variants (TTNtv) in familial and early-onset AF [8], and the association of exonic SNPs in *TTN* with AF in a large GWAS [6] support the hypothesis of an atrial cardiomyopathy [11].

Moreover, our analysis of genetic correlation between AF and other traits (Figure 1) shows a significant genetic correlation between AF and non-ischemic cardiomyopathy (r_g_ = 0.42; *p* = 3 × 10^−4^), further supporting the hypothesis of atrial cardiomyopathy playing a role in AF development.

### 4.4. Treatment Consequences

Catheter ablation of AF is a class I recommendation when antiarrhythmic treatment fails [2]. Several studies showed that AF patients with a higher degree of fibrosis in the atria have higher recurrence rates after catheter ablation [36]. It was, therefore, discussed whether ablative therapy is beneficial when AF occurs as a result of atrial cardiomyopathy [37]. Ongoing studies are evaluating whether detection of fibrosis before ablation, using late gadolinium enhancement magnetic resonance, may improve the ablation of AF in fibrotic hearts [38]. Several of the genes examined in this study are linked with cardiac fibrosis. Cardiac fibrosis is a well-known clinical feature of *DMD*-associated DCM [39], and *PDLIM3* was shown to be involved in cardiac collagen deposition [40].

Finally, these patients might be at higher risk of tachycardia-induced cardiomyopathy in the ventricles, as they are already predisposed for DCM by carrying variants in genes associated with DCM. Understanding the pathogenesis of disease in patients carrying variants in these genes could yield valuable knowledge about the treatment of AF and atrial cardiomyopathy.

### 4.5. Limitations

While the study included a relatively large number of participants, we only identified a total of six patients with LOF variants. Four of these were in the *DMD* gene, likely because of its large size (79 exons) and numerous isoforms, while only two of the variants were in other cytoskeletal genes. Furthermore, most of the individuals with LOF variants were male, including the four carriers of *DMD* variants, who were hemizygous for the variants. It cannot be excluded that these variants, or similar LOF variants in cytoskeletal genes could have other effects in other population groups.

Several of the participants reported family members with AF. However, these individuals were not genetically tested and, therefore, it was not possible to perform co-segregation analyses. The lack of genetic testing of family members is a significant limitation of the study.

Furthermore, our genetic analyses were focused on genes encoding cytoskeletal proteins of cardiomyocytes. These genes only represent a subgroup of structural genes associated with DCM, and the results of this study cannot be extrapolated to all DCM-associated genes.

Therefore, while our data suggest a possible link between LOF variants in structural, cytoskeletal genes and AF, our findings should be regarded as hypothesis-generating and be interpreted with caution.

## 5. Conclusions

We identified six individuals with LOF variants in three cytoskeletal genes in a cohort of 527 early-onset AF patients. We also found significant genetic correlation between AF and non-ischemic cardiomyopathy using publicly available summary statistics from recent genome-wide association studies. Our data suggest that cytoskeletal genes previously associated with ventricular cardiomyopathy may also play a role in AF. Combined with numerous recent studies on other structural genes, these data add to the hypothesis that structural genes could possibly predispose to development of atrial cardiomyopathy.

## Figures and Tables

**Table 1:** Clinical characteristics of populations.

	Early-Onset AF Cohort (*n* = 527)	Control Cohort (*n* = 383)
Sex, male, *n* (%)	441 (83.6)	257 (67)
Age, years, median (IQR)	30 (24–36) ^a^	71 (66–76) ^b^
Height, cm, mean (SD)	183 (±9)	172 (±9)
Weight, kg, mean (SD)	89 (±17)	77 (±15)
BMI, kg/m^2^, mean (SD)	26 (±5)	26 (±5)
**Comorbidities:**		
Hypertension, *n* (%)	0 (0)	246 (64.2)
Diabetes, *n* (%)	0 (0)	39 (10.2)
Heart failure, *n* (%)	0 (0)	0 (0)
Ischemic heart disease, *n* (%)	0 (0)	0 (0)
Valvular heart disease, *n* (%)	0 (0)	0 (0)

AF, atrial fibrillation; BMI, body mass index; IQR, interquartile range; SD, standard deviation. ^a^ Age of AF onset; ^b^ age at enrolment in cohort.

**Table 2:** Clinical characteristics of variant carriers.

Patient	Gene	Variant	RefSNP	Gender	Genotype	Onset of AF (age in years)	AF Type	LVEF (%)	Family History of AF (self-reported)
I	*DMD*	p.D615Efs*6	rs752332058	Male	Hemizygote	28	Persistent	>55	No
II	*DMD*	p.D615Efs*6	rs752332058	Male	Hemizygote	25	Persistent	>55	Yes
III	*DMD*	c.10262+1G > A	rs145603325	Male	Hemizygote	21	Persistent	>55	Yes
IV	*DMD*	c.10262+1G > A	rs145603325	Male	Hemizygote	28	Persistent	>55	No
V	*FKTN*	Chr9:108358933C > T	NA	Male	Heterozygote	31	Paroxysmal	NA	No
VI	*PDLIM3*	Chr4:186425651_186425652del	NA	Female	Heterozygote	40	Paroxysmal	>55	Yes

AF, atrial fibrillation; LVEF, left-ventricular ejection fraction; NA, not available; RefSNP, reference single-nucleotide polymorphism.

**Table 3:** Loss-of-function variants identified in cytoskeletal genes.

Gene	Genomic Position	RefSNP	Transcript	Consequence	Effect	GnomAD MAF (%)
*DMD*	ChrX:31140001_31140013del	rs752332058	ENST00000378723	p.D615Efs*6	Frameshift variant	0.02491
*DMD*	ChrX:31196048C>T	rs145603325	ENST00000357033	c.10262+1G>A	Splice donor	0.02689
*FKTN*	Chr9:108358933C>T	NA	ENST00000223528	p.Q54*	Nonsense variant	NA
*PDLIM3*	Chr4:186425651_186425652del	NA	ENST00000284771	p.C246*fs*1	Frameshift variant	NA

GnomAD, Genome Aggregation Database; MAF, minor allele frequency; NA, not available; RefSNP, reference single-nucleotide polymorphism.

## Supplementary Materials

The following are available online at https://www.mdpi.com/2077-0383/9/2/372/s1: Figure S1: *DMD* isoform expression; Figure S2: *FKTN* isoform expression; Figure S3: *PDLIM3* isoform expression; Table S1: Descriptive information on studies used for genetic correlation analysis of AF and other traits; Table S2: All affected isoforms of *DMD*, *FKTN*, and *PDLIM3*; Table S3: Summary of protein-protein interactions of *DMD*, *FKTN*, and *PDLIM3*.
