# Supplementary material for: Loss-of-Function Variants in Cytoskeletal Genes Are Associated with Early-Onset Atrial Fibrillation
Source: J Clin Med. 2020 Jan 29;9(2):372. doi: 10.3390/jcm9020372 (PMC7074234; doi:10.3390/jcm9020372)
Supplement: Supplementary file 1 [file jcm-09-00372-s001.pdf]

Supplementary Material:

Loss-of-Function Variants in Cytoskeletal Genes Are Associated with Early-Onset Atrial Fibrillation

Oliver Bundgaard Vad [1,2], Christian Paludan-Müller [1,2], Gustav Ahlberg [1,2], Silje Madeleine Kalstø [3], Jonas Ghouse [1,2], Laura Andreasen [1,2], Stig Haunsø [2,4], Arnljot Tveit [3,5], Ahmad Sajadieh [6], Ingrid Elisabeth Christophersen [3,7], Jesper Hastrup Svendsen [2,4] and Morten Salling Olesen [1,2]

1. Department of Biomedical Sciences, Faculty of Health and Medical Sciences, University of Copenhagen, 2200 Copenhagen N, Denmark;
2. Laboratory for Molecular Cardiology, Department of Cardiology, Centre for Cardiac, Vascular, Pulmonary and Infectious Diseases, Rigshospitalet, Copenhagen University Hospital, 2100 Copenhagen O, Denmark;
3. Department of Medical Research, Bærum Hospital, Vestre Viken Hospital Trust, 3004 Drammen, Norway;
4. Department of Clinical Medicine, Faculty of Health and Medical Sciences, University of Copenhagen, 2200 Copenhagen N, Denmark;
5. Department of Cardiology, Institute of Clinical Medicine, University of Oslo, 0450 Oslo, Norway;
6. Department of Cardiology, Copenhagen University Hospital, Bispebjerg, 2400 Copenhagen NV, Denmark;
7. Department of Medical Genetics, Oslo University Hospital, 0450 Oslo, Norway;

*Correspondence: mortensol@sund.ku.dk; Tel.: +4535330753

Table of contents:

Table S1 Descriptive information on studies used for genetic correlation analysis of AF and other traits 3

Table S2 All affected isoforms of *DMD, FKTN*, and *PDLIM3*........4

Table S3 Summary of protein-protein interactions of *DMD*, *FKTN*, and *PDLIM3*. 5

Figure S1 *DMD* isoform expression........6

Figure S2 *FKTN* isoform expression 7

Figure S3 *PDLIM3* isoform expression........8

References ....**Error! Bookmark not defined.**

Table S1 Descriptive information on studies used for genetic correlation analysis of AF and other traits.

Trait	Study	No. of participants:	Ancestry of participants	Reference
Alcohol dependence	Trans-ancestral GWAS of alcohol dependence reveals common genetic underpinnings with psychiatric disorders	28,757	European	(1)
Angina	www.nealelab.is/uk-biobank/	337,159	European	(2)
BMI	www.nealelab.is/uk-biobank/	336,107	European	(2)
Coronary heart disease	Identification of 64 novel genetic loci provides an expanded view on the genetic architecture of coronary artery disease	400,961	European	(3)
Depression	Meta-analysis of genome-wide association studies for neuroticism in 449,484 individuals identifies novel genetic loci and pathways	357,957	European	(4)
Diabetes type 2	Large-scale association analysis provides insights into the genetic architecture and pathophysiology of type 2 diabetes	69,033	European	(5)
Ever smoked	www.nealelab.is/uk-biobank/	336,067	European	(2)
Hand grip strength	www.nealelab.is/uk-biobank/	335,821	European	(2)
Heart failure	Phenotypic refinement of heart failure in a national biobank facilitates genetic discovery	394,156	European	(6)
Height	www.nealelab.is/uk-biobank/	336,474	European	(2)
Hypertension	www.nealelab.is/uk-biobank/	361,194	European	(2)
Non-ischemic cardiomyopathy	Phenotypic refinement of heart failure in a national biobank facilitates genetic discovery	390,142	European	(6)
Overall health rating	www.nealelab.is/uk-biobank/	336,020	European	(2)

Table S2. All affected isoforms of *DMD*, *FKTN*, and *PDLIM3*.

Gene	Genomic Position	Amino-Acid Change	RefSNP	Effect	Transcript	Consequence
DMD	ChrX:31140001_31140013	delTCTGCCCAAATCA	rs752332058	Frameshift variant	ENST00000343523	p.D1113Efs*6
					ENST00000358062	p.D1366Efs*6
					ENST00000359836	p.D1210Efs*6
					ENST00000361471	p.D602Efs*6
					ENST00000378680	p.D505Efs*6
					ENST00000378723	p.D615Efs*6
					ENST00000474231	p.D1223Efs*6
DMD	ChrX:31196048	C>T	rs145603325	Splice donor	ENST00000357033	c.10262+1G > A
					ENST00000378677	c.10250+1G > A
					ENST00000378702	c.1058+1G > A
					ENST00000378705	c.632+1G > A
					ENST00000378707	c.2882+1G > A
					ENST00000378723	c.1058+1G > A
					ENST00000474231	c.2882+1G > A
FKTN	Chr9:108358933	C>T	NA	Nonsense variant	ENST00000223528	p.Q54*
					ENST00000357998	p.Q54*
					ENST00000448551	p.Q54*
					ENST00000540160	p.Q54*
					ENST00000602661	p.Q54*
PDLIM3	Chr4:186425651_186425652	delCA	NA	Frameshift variant	ENST00000284770	p.C294*fs*1
					ENST00000284771	p.C246*fs*1

Table S3. Summary of protein-protein interactions of *DMD*, *FKTN*, and *PDLIM3*.

Gene	Interaction (confidence score)	Gene	Interaction (confidence score)	Gene	Interaction (confidence score)
DMD	*DAG1* (0.997)	*FKTN*	*FKRP* (0.992)	*PDLIM3*	*NEB* (0.877)
DMD	*SNTG2* (0.989)	*FKTN*	*ISPD* (0.991)	*PDLIM3*	*TNNC2* (0.875)
DMD	*SNTB1* (0.987)	*FKTN*	*POMK* (0.977)	*PDLIM3*	*PDLIM7* (0.873)
DMD	*CAV3* (0.987)	*FKTN*	*PO*MGNT1 (0.958)	*PDLIM3*	*MYL1* (0.866)
DMD	*SGCD* (0.986)	*FKTN*	*POMT1* (0.952)	*PDLIM3*	*CAV3* (0.852)
DMD	*SNTA1* (0.982)	*FKTN*	*POMT2* (0.950)	*PDLIM3*	*MYBPC3* (0.810)
DMD	*SGCB* (0.979)	*FKTN*	*DAG1* (0.870)	*PDLIM3*	*ACTN2* (0.789)
DMD	*LAMA2* (0.977)	*FKTN*	*THEM5* (0.852)	*PDLIM3*	*SRL* (0.705)
DMD	*AGRN* (0.975)	*FKTN*	*POMGNT2* (0.840)		
DMD	*TTN* (0.972)	*FKTN*	*DPM3* (0.824)		

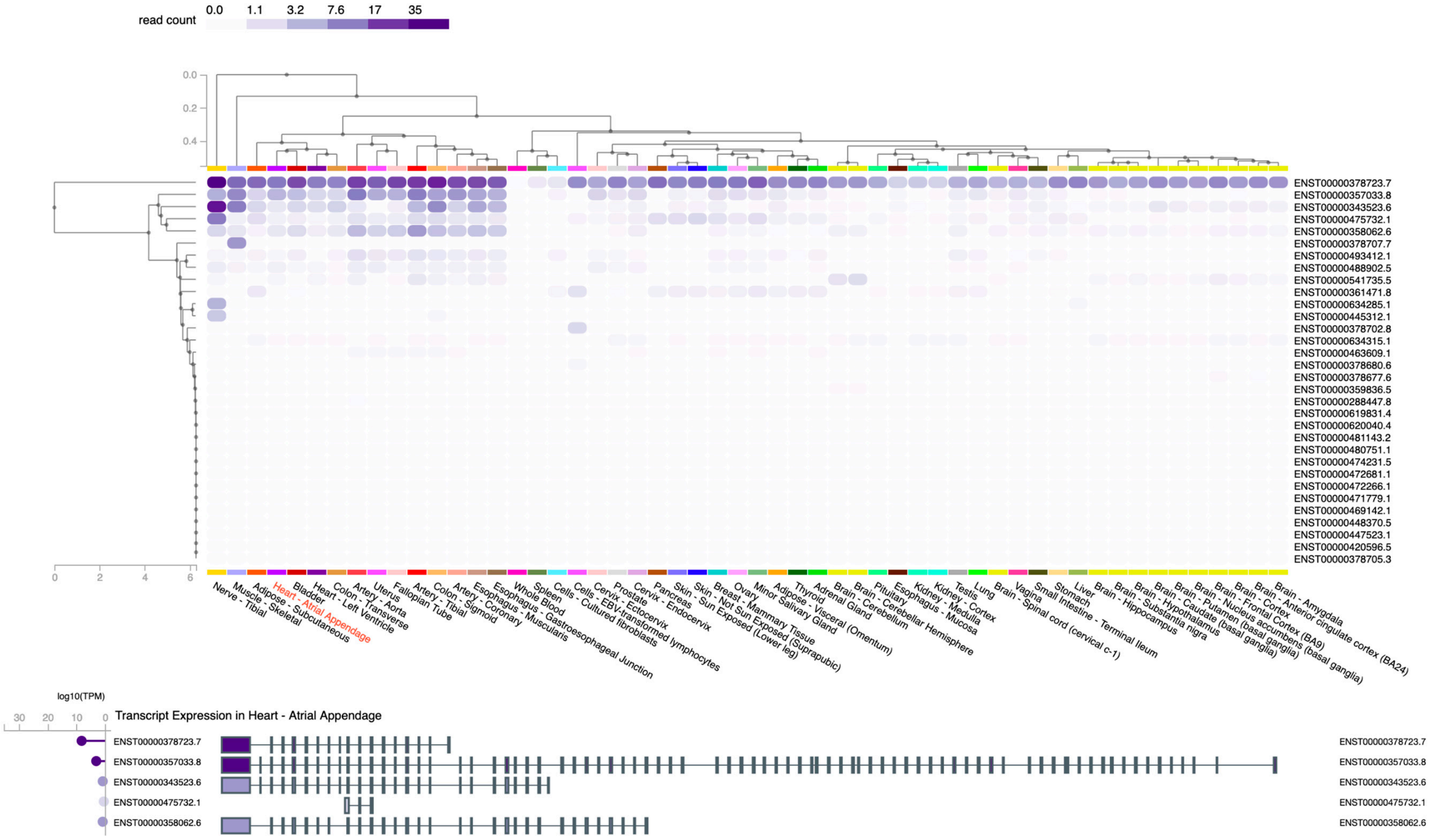

Figure S1. *DMD* isoform expression.

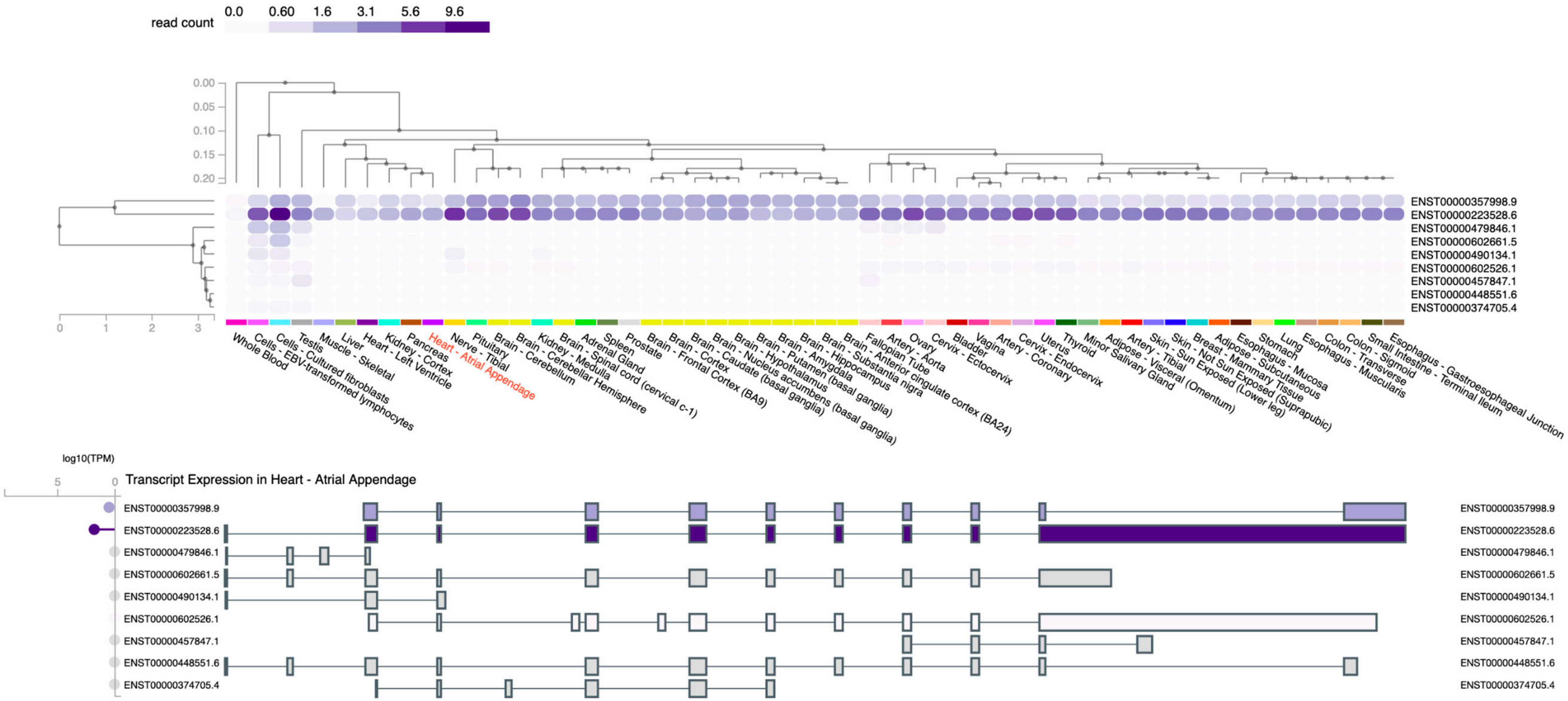

Figure S2. *FKTN* isoform expression.

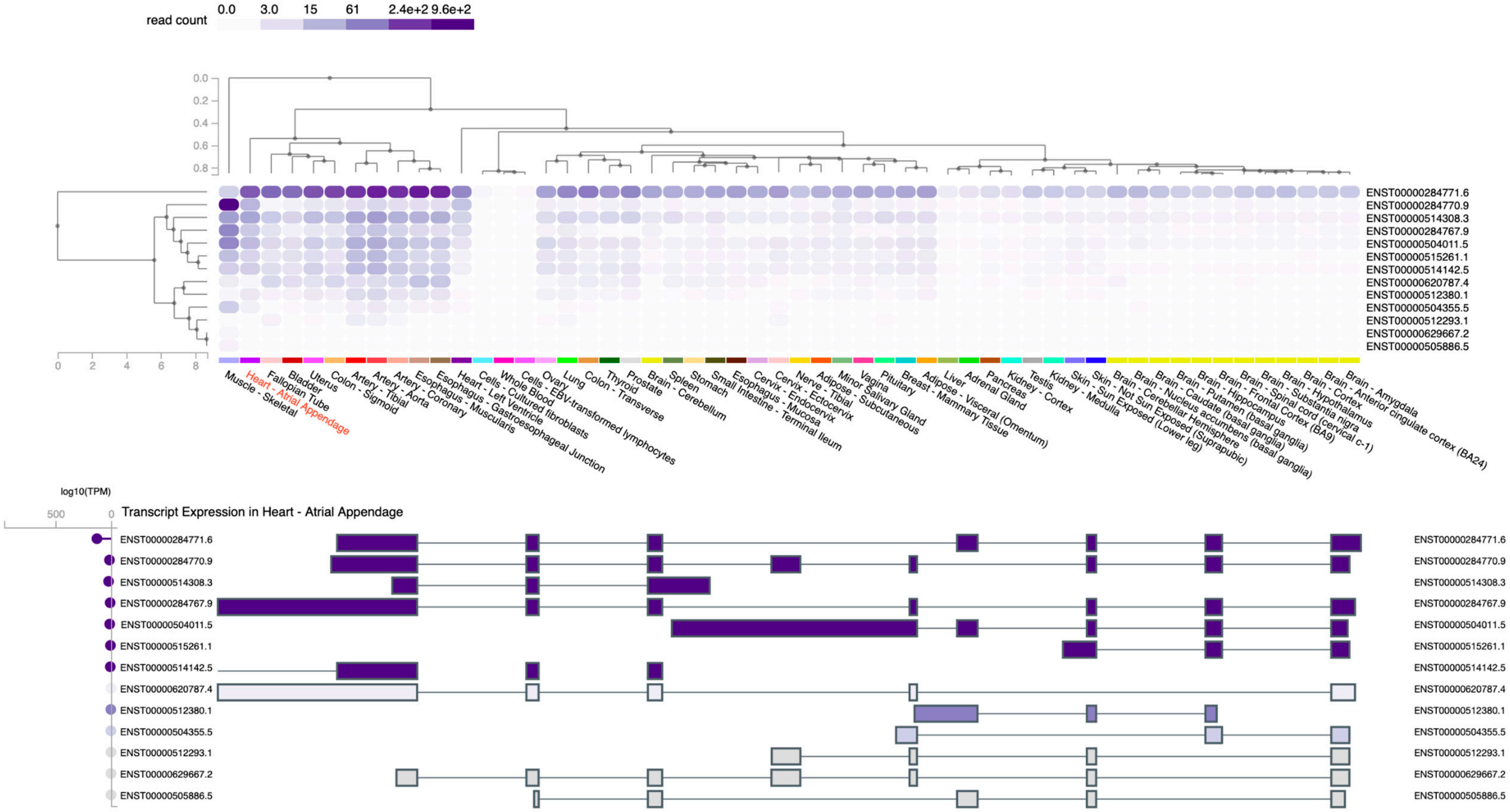

Figure S3. *PDLIM3* isoform expression.

References

1. McGue, M.; Zhang, Y.; Miller, M.B.; Basu, S.; Vrieze S.; Hicks, B.; Malone, S.; Oetting, W.S.; Iacono, W.G. A genome-wide association study of behavioral disinhibition. *Behav Genet.* **2013** Sep; *43* (5): 363–73.
2. UK Biobank [Internet]. Neale lab. [cited 2019 Dec 9]. Available from: http://www.nealelab.is/uk-biobank
3. van der Harst P, Verweij N. Identification of 64 Novel Genetic Loci Provides an Expanded View on the Genetic Architecture of Coronary Artery Disease. *Circ Res*. **2018** 02; *122*(3): 433–43.
4. Nagel, M.; Jansen, P.R.; Stringer, S.; Watanabe, K.; de Leeuw, C.A.; Bryois, J.; Savage, J.E.; Hammerschlag, A.R.; Skene, N.G.; Muñoz-Manchado, A.B; et al. Meta-analysis of genome-wide association studies for neuroticism in 449,484 individuals identifies novel genetic loci and pathways. *Nat Genet.* **2018** *50* (7): 920–7.
5. Morris, A.P.; Voight, B.F.; Teslovich, T.M.; Ferreira, T.; Segrè, A.V; Steinthorsdottir, V.; Strawbridge, R.J.; Khan, H.; Grallert, H.; Mahajan, A.; et al. Large-scale association analysis provides insights into the genetic architecture and pathophysiology of type 2 diabetes. *Nat Genet*. **2012** Sep; *44* (9): 981–90.
6. Aragam, K.G.; Chaffin, M.; Levinson, R.T.; McDermott, G.; Choi, S-H.; Shoemaker, M.B.; Haas, M.E.; Weng, L-C.; Lindsay, M.E.; Smith, J.G.; et al. Phenotypic Refinement of Heart Failure in a National Biobank Facilitates Genetic Discovery. *Circulation.* **2018** Nov 11;
